# Research Progress of Fecal Microbiota Transplantation in Liver Diseases

**DOI:** 10.3390/jcm12041683

**Published:** 2023-02-20

**Authors:** Yuanyuan Zhao, Chen Gong, Jing Xu, Dong Chen, Bo Yang, Zhishui Chen, Lai Wei

**Affiliations:** 1 Institute of Organ Transplantation, Tongji Hospital, Tongji Medical College, Huazhong University of Science and Technology; Key Laboratory of Organ Transplantation, Ministry of Education; NHC Key Laboratory of Organ Transplantation; Key Laboratory of Organ Transplantation, Chinese Academy of Medical Sciences, Wuhan 430030, China; 2 Department of Oncology, Tongji Hospital, Tongji Medical College, Huazhong University of Science and Technology, Wuhan 430030, China

**Keywords:** fecal microbiota transplantation, chronic liver diseases, gut microbiota, gut-liver axis, liver transplantation

## Abstract

A growing body of evidence suggested that gut microbiota is associated with liver diseases through the gut–liver axis. The imbalance of gut microbiota could be correlated with the occurrence, development, and prognosis of a series of liver diseases, including alcoholic liver disease (ALD), non-alcoholic fatty liver disease (NAFLD), viral hepatitis, cirrhosis, primary sclerosing cholangitis (PSC), and hepatocellular carcinoma (HCC). Fecal microbiota transplantation (FMT) seems to be a method to normalize the patient’s gut microbiota. This method has been traced back to the 4th century. In recent decade, FMT has been highly regarded in several clinical trials. As a novel approach to reconstruct the intestinal microecological balance, FMT has been used to treat the chronic liver diseases. Therefore, in this review, the role of FMT in the treatment of liver diseases was summarized. In addition, the relationship between gut and liver was explored through the gut–liver axis, and the definition, objectives, advantages, and procedures of FMT were described. Finally, the clinical value of FMT therapy in liver transplant (LT) recipients was briefly discussed.

## 1. Introduction

As a novel approach to reconstruct the intestinal microecological balance, fecal microbiota transplantation (FMT) has been gradually and widely practiced in the treatment of a variety of diseases in recent decade. This method transfers processed fecal materials from healthy donors to patients to rebuild the balance of their gut microbiota [1]. Under normal conditions, the human gastrointestinal (GI) tract, which is colonized with numerous bacterial species, individually differs and is relatively stable over time [2], while several genetic backgrounds and environmental factors, such as diet, viruses, and use of drugs can alter the balance and further cause a variety of diseases [3,4]. On the other hand, some diseases (e.g., chronic liver diseases) can also break the balance of gut microbiota. In 2013, Els et al. performed the first randomized controlled trial and demonstrated that duodenal infusion of donor feces into patients with Clostridium difficile infection (CDI) had a significant efficacy in resolving symptoms than use of antibiotics alone [5]. To date, FMT has earned endorsement of professional societies in the treatment of antibiotic-refractory CDI [6,7]. In addition, FMT has been applied to treat other diseases, such as autoimmune diseases, behavioral diseases, metabolic disorders, and organic diseases.

In fact, a great number of studies demonstrated that gut microbiota is associated with liver diseases. In 1987, it was first found that the relationship between the gut and liver was bidirectional and a cyclic process, and this physiological process was described as the gut–liver axis [1]. Accordingly, in recent two decades, researchers demonstrated that prebiotics could alleviate fat mass development and associated hepatic steatosis. Moreover, the imbalance of gut microbiota could result in the occurrence and development of a series of liver diseases, including alcoholic liver disease (ALD), non-alcoholic fatty liver disease (NAFLD), viral hepatitis, cirrhosis, primary sclerosing cholangitis (PBC), hepatocellular carcinoma (HCC), and even hepatic encephalopathy (HE) [8,9,10,11,12,13]. Based on these backgrounds, FMT has been increasingly applied in various types of chronic liver diseases [14]. As a whole, the use of FMT in liver diseases is still in the initial stage. The present review aimed to mainly concentrate on the application of FMT in the treatment of liver diseases. In addition, the relationship between gut and liver was explored through the gut–liver axis, and the definition, objectives, advantages, and technical process of FMT were described. Finally, the clinical value of FMT in liver transplant (LT) recipients was briefly discussed.

## 2. The Gut Microbiota and the Gut–Liver Axis

The gut microbiota consists of millions of species, with weight of approximately 1–2 kg [15,16]. The gut microbiota has been considered as an indispensable “organ” [17]. In recent twenty years, the advent of genetic tools and the metagenomics assisted scholars to realize the composition and function of gut microbiota and their association with several potential diseases. The gut microbiota has important functions in hormonal responses, inflammatory pathways, immune reactions, and metabolites [18].

**Figure 1:**
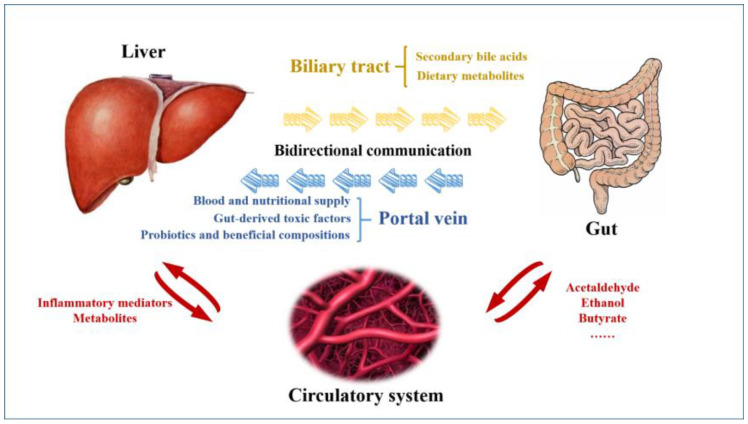
**The diagrammatic representation of the gut–liver axis.** The gut–liver axis refers to the bidirectional communication between the gastrointestinal tract and the liver by the biliary tract, the portal vein, and systemic circulation. Through portal vein, liver mainly receives blood and nutritional supply, as well as gut-derived toxic factors. Probiotics and beneficial compositions from the gut can also protect liver through the gut–liver axis. Liver regulates the intestinal function and balance of gut microbiota through the bile acid.

To date, alteration of gut microbiota has been reported to be associated with numerous liver diseases, which could be related to the existence of the gut–liver axis (Figure 1). Generally, the gut–liver axis refers to the bidirectional communication between GI tract and liver by biliary tract, portal vein, and systemic circulation [19]. Through portal vein, liver mainly receives almost two-thirds of its blood and nutritional supply from the gut, as well as gut-derived toxic factors, such as metabolites, damage/pathogen-associated molecular patterns, and detrimental microbiota [20]. These detrimental factors stimulate hepatocytes and hepatic immune cells, activate inflammation-related pathways, and finally cause liver diseases. On the one hand, probiotics and beneficial compositions from the gut can protect liver through the gut–liver axis [21]. On the other hand, liver regulates the intestinal function and balance of gut microbiota through circulation of bile acid. Therefore, the close interaction between gut and liver may be a very important factor in the pathogenesis of liver diseases.

## 3. Fecal Microbiota Transplantation (FMT)

### 3.1. Definition, Objectives, and Advantages

FMT is also known as stool transplantation. FMT is a procedure, in which stool from a healthy donor is placed into another patient’s GI tract [22]. The components of the fecal transplants contain about 55% of microbiota and 24% of soluble components, including mucus, fat, proteins, small molecules, short chain fatty acids, etc. [23]. To date, FMT has been widely used in the treatment of recurrent CDI [24]. In addition, with the fast development of high-throughput sequencing technologies, a variety of diseases, such as diabetes, various types of cancer, and organ diseases were found to be associated with the gut microbiota [25]. Compared with using antibiotics to eliminate specific pathogenic strains and administration of specific probiotics, FMT can transfer a more complete and stable fecal microbial community of gut micro-organisms [26]. Therefore, FMT possesses greater advantages. After allogeneic organ transplantation, rejection may be caused by the immune system, triggering a response that will ultimately destroy the transplanted organ or tissue. FMT can also be a type of transplantation, while it does not exist as an immune response, which differs from other types of organ transplantation.

### 3.2. Preparation and Delivery

For FMT, fecal stools are mainly obtained from selected healthy donors, which can restore the balance of healthy microbiota and facilitate clear infection for patients. Before FMT, fecal stools need to be processed and prepared, and they can be then transplanted into recipients [25]. The processes of FMT preparation and delivery are briefly discussed in the following sections.

**Figure 2:**
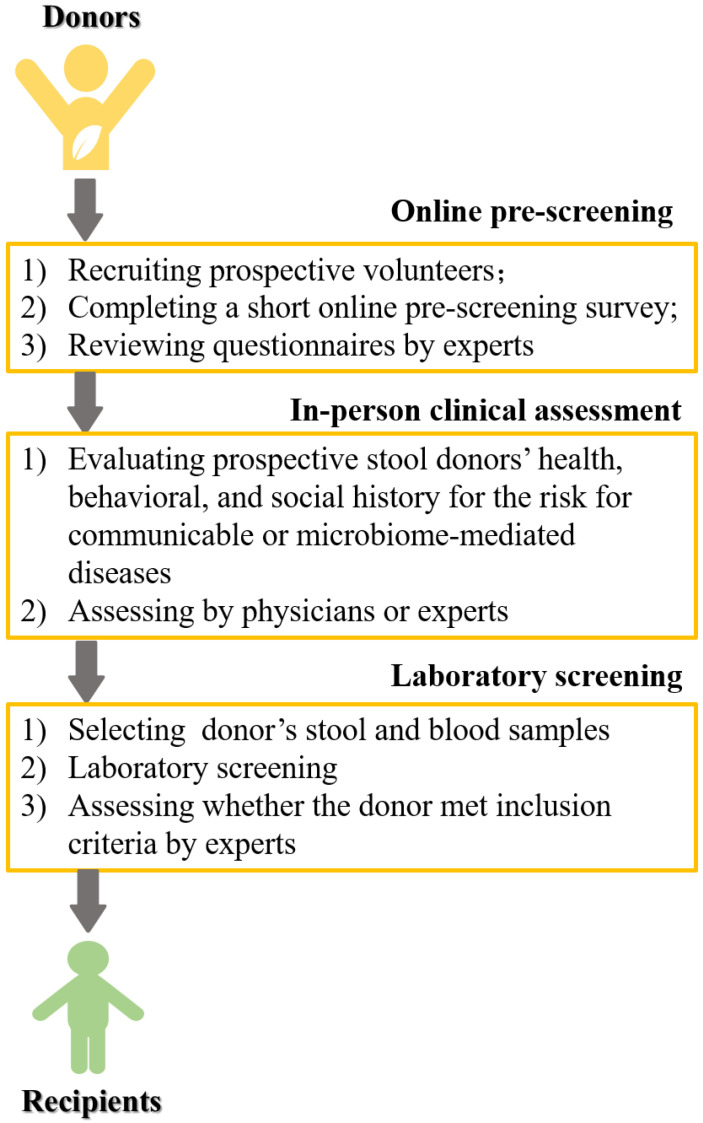
The process of FMT donor selection.

In fact, similar to organ transplantation, fecal stools for FMT are mainly obtained from healthy and selected donors. Before the emergence of stool banks, finding and screening eligible donors were challenging [27]. Stool banks have recently emerged, while their availability is limited. Generally, the process of donor screening includes online pre-screening, clinical assessment, and laboratory screening [28] (Figure 2). Before being transplanted into patients or recipients, fecal stools need to be processed and prepared. The commonly used methods have been described in Biazzo et al.’ review [25], while the detailed methods vary among different studies. In the stage of sample preparation, after obtaining stool samples from healthy donors, they mainly need to be homogenized and liquefied by blending with sterile saline. Afterwards, residual solid feces are filtered out through metal sieve. Then, homogenous liquid samples can be achieved. In the stage of microbiota preparation, fresh stools are transferred into frozen stools. Finally, these treated samples are kept at −80 °C for later FMT. When patients need FMT, frozen samples are thawed at 4 °C and reconstituted with normal saline. It is noteworthy that the standards are not widely accepted for the quality and safety control of microbiota. A previous study reported a female case who underwent FMT for recurrent and developed new-onset obesity after receiving stool from a healthy overweight donor [29]. Therefore, additional clinical studies should be performed to acquire more reliable evidence about the standard of FMT.

## 4. Fecal Microbiota Transplantation (FMT) in the Treatment of Liver Diseases

As mentioned earlier, it was confirmed that the gut–liver axis plays an important and critical role in progression of liver diseases. Disturbances in the intestinal barrier may increase the portal influx of bacteria and their products into the liver, and further worsen a range of hepatic diseases [30,31]. Recently, a growing body of evidence demonstrated that dysfunction of gut microbiota plays a key role in the pathogenesis of ALD and NAFLD. Furthermore, other liver-associated infections, autoimmune hepatitis (AIH), and HCC have been demonstrated to be caused by dysfunction of gut microbiota. In the present study, application of FMT in the treatment of liver diseases was reviewed and discussed. The clinical trials were summarized in Table 1.

### 4.1. Hepatitis B Virus (HBV) Infection

HBV infection is one of the most common public health challenges in the world, and about 15–40% of HBV-infected patients may finally develop chronic liver diseases, including cirrhosis, liver failure, and even HCC [32]. The ideal endpoint of HBV-infected patients is hepatitis B surface antigen (HBsAg) loss [33]. For HBV e-antigen (HBeAg)-positive chronic hepatitis B (CHB) patients, HBeAg seroconversion is mainly the first step for treatment [34]. With the significant advances in the treatment of HBV infection, several approved therapies, including oral nucleos(t)ide analogue(s)—entecavir (ETV), tenofovir disoproxil fumarate (TDF)/tenofovir, alafenamide (TAF), and peg-interferon can be used [35]. Despite using these methods, only few patients could obtain HBeAg clearance or seroconversion, even after multiple years of antiviral therapy [36]. The specific reason has not been clearly expounded. Over the past few years, some studies demonstrated that recent therapies neglected the role of gut microbiota, and it may play a key role in immune clearance of HBV [37]. Several pilot trials with the small sample size have been conducted to explore the therapeutic effects of FMT on CHB patients. In 2017, Ren et al. first carried out a case-controlled, open-label pilot study on the application of FMT in 18 CHB patients who remained HBeAg-positive, following >3 years of ongoing ETV- or TDF-based antiviral therapy [36]. Among them, 5 patients were included in the FMT arm who received 1–7 cycles of FMT, and 40% (2/5) of patients achieved HBeAg clearance after 1–2 cycles of FMT. In 2021, Chauhan et al. performed another similar pilot study, and their results showed that in the FMT arm, 16.7% (2/12) of patients had HBeAg clearance. While in the AVT arm, no patient achieved HBeAg clearance [35]. These clinical studies confirmed the significant effects of FMT on stubborn CHB patients. However, more evidence from large-scale prospective studies is required.

Several studies attempted to explain the mechanism underlying whether the gut microbiota composition could affect the HBV infection. The CHB infection has been found to be associated with the dysfunction of HBV-specific immune responses, causing failure in the treatment of infected hepatocytes [38]. Using animal models, some risk factors, such as use of antibiotics, have been demonstrated to impair gut barrier function and increase gut permeability, leading to commensal bacterial translocation from the gut to the liver, suppressed T-cell response in the liver, and prolonged HBV infection [39]. In addition, the genetic background is a key factor in determining outcomes of patients with HBV infection. Wang et al. found that HBV infection was only persisted in C57BL/6J mice, rather than in C57BL/6N mice [40], and another study showed that in different strains of mice, duration of HBV infection was significantly different [41]. Furthermore, it has been reported that HBV infection could alter the intestinal microbiota. For instance, compared with healthy controls, the levels of Bifidobacteria and Lactobacillus were higher, and the levels of Enterococcus and Enterobacteriaceae were lower in CHB patients [42]. Moreover, compared with healthy controls, Enterobacteriaceae, Faecalibacterium prausnitzii, and Enterococcus faecalis showed a noticeable increase in asymptomatic HBV carriers, and the increased range was significantly greater in HBV patients [43]. These changes caused an increase in bacterial translocation and endotoxin load, in which activation of Toll-like receptor (TLR) facilitated immune-mediated liver injury.

### 4.2. Non-Alcoholic Fatty Liver Disease (NAFLD)

NAFLD is one of the most common liver diseases, influencing 10–24% of world’s population [18]. NAFLD includes simple steatosis, nonalcoholic steatohepatitis (NASH), more severe cirrhosis as end-stage organ failure, and HCC. Recent studies have demonstrated that NAFLD was associated with a series of metabolic diseases, including obesity, type 2 diabetes mellitus (T2DM), and even cancer [44,45,46]. FMT has been reported as a potential therapeutic method for several metabolic diseases. In 2013, Roy et al. first found that NAFLD could be transmitted by FMT in mice [47]. Subsequently, Zhou et al. established a mouse model of high-fat diet (HFD)-induced steatohepatitis. After an eight-week HFD, FMT was carried out for eight weeks. This experiment demonstrated that the bacterial antigen translocation caused systemic inflammation in NAFLD patients [48]. FMT intervention could correct the gut microbiota disturbance and reverse steatohepatitis in mice fed with HFD [49]. In addition, Gomez-Hurtado et al. concentrated on the application of FMT in patients with NAFLD [50]. In this randomized clinical trial, a total of 47 patients with NAFLD were randomly assigned to FMT group who received FMT from healthy donors. Compared with patients in the non-FMT group (N = 28) who received original treatment, the clinical symptoms of NAFLD in patients undergoing FMT were significantly improved, confirming that the FMT has potential therapeutic effects on NAFLD. In addition, numerous ongoing trials concentrated on FMT and its potential in NAFLD and NASH (Identifiers: NCT02496390 and NCT02469272, respectively).

**Figure 3:**
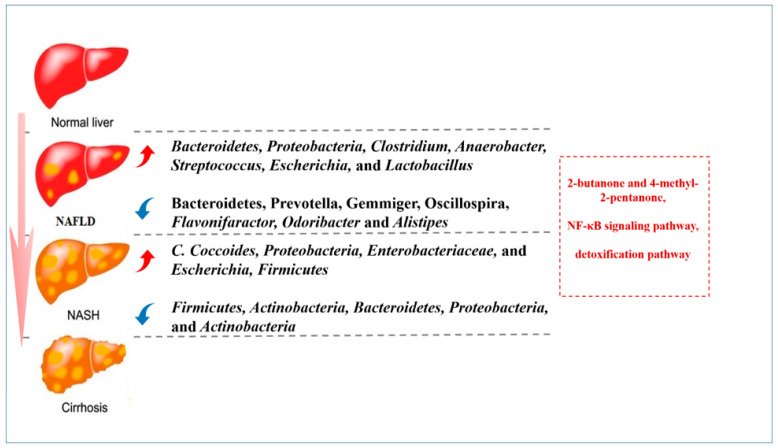
**The potential signaling pathways and alteration of gut microbiota in patients with NAFLD/NASH.** The figure shows the alteration of gut microbiota and potential signaling pathways in the process from normal liver to NAFLD/NASH, and finally to cirrhosis.

The potential mechanism indicating how the gut microbiota could affect NAFLD has been widely discussed in several studies (Figure 3). It has been reported that patients with NAFLD had significantly increased levels of Clostridium, Anaerobacter, Streptococcus, Escherichia, and Lactobacillus, and reduced levels of Flavonifaractor, Odoribacter, Alistipes, etc. [51]. While in patients with NASH, the levels of Proteobacteria, Enterobacteriaceae, Escherichia, etc., were higher [52]. These alterations can induce the levels of 2-butanone and 4-methyl-2-pentanone, which are associated with hepatocellular toxicity [53]. Additionally, a review expounded that enrichment of ethanol-producing bacteria could cause abundant ethanol in the body of patients with NALFD, which could activate nuclear factor-κB (NF-κB) signaling pathway and cause liver damage [54]. Furthermore, detoxification pathway was weakened in patients with NALFD, which could cause oxidative injury to the hepatocytes and induce inflammation and steatohepatitis [55].

### 4.3. Alcoholic Liver Disease (ALD)

ALD is a spectrum of diseases, ranging from asymptomatic liver steatosis to the development of fibrosis, cirrhosis, and alcoholic hepatitis [56]. According to epidemiological data, about 20–30% of patients with a history of alcohol misuse progress to liver damage, and even liver cirrhosis or alcoholic hepatitis [57]. Recently, ensuring lasting alcohol abstinence is the key to prevent the occurrence and development of ALD. Only one-third of patients with alcoholic hepatitis are eligible for steroid therapy. In the late stage of ALD, LT is necessary, while it remains controversial whether it may be effective in the risk of infections, post-transplant recurrence, and long-time transplant waitlist. Over the past few years, studies demonstrated that gut microbiota played a key role in the progression of ALD [58,59]. Ciocan et al. enrolled patients with various degrees of ALD and assessed their structure of intestinal microbiota and function in bile acid homeostasis. They found that patients with cirrhosis and ALD had a higher level of total plasma bile acid, whereas levels of total and secondary bile acids in these cases were lower than those in healthy donors. Furthermore, ALD patients had a higher abundance of Actinobacteria and a lower level of Bacteroidetes [60]. Bajaj et al. summarized investigations into ALD and microbiota composition and function in humans in a review on ALD and the gut microbiota [56]. Using this review it was confirmed that patients with different degrees of ALD have varying structures and levels of microbiota, proving a close association between the gut microbiota and ALD. Moreover, numerous studies revealed the special mechanism indicating how the specific intestinal microbiota could affect ALD. For instance, using a mouse model of ALD, Wrzosek et al. found that tryptophan metabolism induced aryl hydrocarbon receptor activation and improved alcohol-induced liver injury [61]. In addition, ursolic acid has been reported to ameliorate intestinal oxidative stress and barrier dysfunction induced by alcohol [62].

According to these positive results from animal studies, in the last five years, some scholars attempted to certify whether FMT could treat ALD in clinic. In 2017, Philips et al. first reported a patient with severe alcohol hepatitis who was a steroid non-responder and underwent FMT [63]. In this case, the patient’s clinical, biochemical, and liver disease severity scores were significantly improved after FMT, which demonstrated that a distinct bacterial population changed before and after FMT. Subsequently, another open-label study was performed with follow-up of 3 months to compare the outcomes in patients with severe alcoholic hepatitis using different methods, including nutritional therapy (n = 17), corticosteroid therapy (n = 8), pentoxifylline therapy (n = 10), and FMT (n = 16) from healthy donors [64]. This clinical trial finally indicated that FMT for severe alcoholic hepatitis could improve survival beyond what is suggested by other therapies. After 1–2 years, the relative abundance of Porphyromonas was significantly lower and that of Bifidobacterium was higher in patients who underwent FMT than in patients who underwent corticosteroid therapy [65]. Furthermore, FMT could function as a cost-effective bridge to LT or to improve survival without transplantation. FMT was also demonstrated as a safe therapeutic approach to reduce the incidence of ALD [66]. Based on the clinical evidence, FMT is suggested as a safe and efficient therapy for ALD, especially for non-corticosteroid-responsive patients and without history of undergoing LT.

### 4.4. Autoimmune Hepatitis (AIH)

AIH is an entity of chronic and immune-mediated hepatitis characterized by hepatocyte injury, with the presence of circulating autoantibodies and elevated level of serum immunoglobulin G (IgG) [67]. AIH can occur globally in all ethnicities and affect both children and adults with an increasing morbidity [68]. At present, the main pathogenetic mechanism of AIH is considered as a loss of tolerance against the patient’s own liver antigens, which is potentially triggered by both genetic and environmental risk factors, such as xenobiotics and pathogens [69,70]. AIH may develop to liver cirrhosis and HCC, and it may even lead to fulminant hepatic failure. AIH can be classified into juvenile AIH (including AIH-1 and AIH-2) and adult AIH (mainly AIH-1) on the basis of the age profile [68]. In fact, triggers of AIH are very complex and have not yet been identified. For treatment, AIH patients favorably respond to corticosteroids, while some patients who are irresponsive to standard treatment may quickly develop to fibrosis and cirrhosis [71]. Therefore, development of effective therapies for patients who are irresponsive to corticosteroids is essential.

In recent years, evidence from murine models exhibited that the gut microbiota is an important environmental risk factor, participating in the pathogenesis of AIH [72,73,74]. Wei et al. performed a cross-sectional study on 91 patients with AIH and 98 healthy controls by 16S rRNA gene sequencing, and the results showed that compared with healthy controls, the gut microbiome of patients with AIH before steroid treatment was accompanied by a lower alpha-diversity and a distinct microbial composition [75]. The research provided evidence for compositional and functional alterations of gut microbiome in AIH, which suggested the potential of using gut microbiota as a biomarker to assess the incidence of AIH. In addition, some similar studies also proved that gut microbiota dysfunction had a functional association with the incidence of AIH [76,77]. Accordingly, Liang et al. established a mouse model of AIH to analyze the therapeutic effects of FMT [78]. This study showed that FMT could modulate imbalance of T follicular regulatory (TFR) and T follicular helper (TH) cells and gut microbiota composition by immune pathways in vivo. Furthermore, metabolite pathways (e.g., short-chain fatty acid, amino acid, and bile acid) and receptor pathways (e.g., Toll-like receptor 4 (TLR4), and G protein-coupled receptors (GRP41/GPR43, GPR109a) in the intestine, while nucleotide-binding and oligomerization domain NOD-like receptors (NLRs), TLR4, TLR9, and GPBAR1 in liver) were found as the influential mechanisms of altered gut microbiota in AIH [79]. The above-mentioned evidence verified the underlying therapeutic value of FMT for AIH. However, to our knowledge, no clinical study has yet assessed the therapeutic value of FMT for AIH.

### 4.5. Primary Sclerosing Cholangitis (PSC)

PSC is a chronic immune-related cholestatic liver disease, which can lead to cholestasis, bile duct stenosis, and hepatic fibrosis [80]. A previous study demonstrated that PSC is closely associated with inflammatory bowel disease (IBD) [81], suggesting that gut microbiota plays a key role in the PSC. Compared with healthy controls, PSC and PSC-IBD patients have significantly distinct gut microbial profiles with decreasing expression of Prevotella copri (P. copri) [82]. Several studies demonstrated that P. copri could improve glucose homeostasis with GI resection by enhancing the bile acid metabolism and signaling [83], and promote immune tolerance [84]. On the other hand, genome-wide association studies have identified that some loci are correlated with both microbiome composition and PSC, including fucosyltransferase 2 (FUT2) [85]. The protective role of gut microbiota in PSC has been demonstrated using germ-free multidrug resistance 2 knockout (mdr2^−/−^) mouse models [86]. Of these, the gut microbiota has emerged as a key environmental risk factor for PSC as an inflammatory disease [87].

For the treatment of PSC, there is no effective therapy, and LT seems to be the only therapeutic option [88]. However, there is still a risk of recurrent PSC after LT. According to the protective role of gut microbiota in PSC, over the past few years, additional studies have concentrated on the therapeutic effects of gut microbiota on PSC. In addition to oral antibiotics (vancomycin, metronidazole, and minocycline), few studies have assessed the therapeutic effects of FMT on PSC. In 2019, Allegretti et al. performed an open-label pilot study on 10 patients with PSC-IBD. These patients underwent FMT, and 30% of them experienced a more than 50% decrease in alkaline phosphatase (ALP) levels. Moreover, no relevant adverse event occurred [89]. Philips et al. reported a single case which received FMT for recurrent bacterial cholangitis in PSC [90]. Following FMT, the patient’s liver biochemistry, bile acid, and bacterial community were significantly improved, suggesting the applicability of FMT in the treatment of PSC. However, further evidence is required to verify the above-mentioned findings.

**Table 1:** Studies related to the application of FMT in the treatment of liver diseases (without case reports).

Diseases	Clinical Trials (Published)	Participants	Outcomes
HBV infection	[35]	AVT: 15 FMT: 12	(1) In the FMT arm, 16.7% (2/12) patients had HBeAg clearance in comparison to none in the AVT arm (*p =* 0.188). (2) None of the patients in either arm had HBsAg loss. (3) The FMT was tolerated well, 42.8% (6/14) patients reported one or more minor adverse events.
	[36]	Control: 13 FMT: 5	(1) A significant decline in HBeAg titer rather than HBsAg titer was observed in the FMT arm compared to that at the baseline. HBeAg titer declined gradually after each time of FMT. (2) None of the control patients achieved HBeAg clearance (0/13) at the conclusion of the study. (3) ALT and HBV DNA were also detected at 4 weeks after each time of FMT and remained under the lower limit of detection. (4) No HBeAg seroconversion was observed at the end of follow-up.
NAFLD	[51]	Oral probiotics: 28 FMT: 47	(1) FMT can decrease the fat accumulation in the liver by improving the gut microbiota dysbiosis. (2) Significant differences in the clinical features and gut microbiota between lean and obese NAFLD patients. (3) FMT had better effects on gut microbiota reconstruction in lean NAFLD than in obese NAFLD patients
ALD	[65]	Treated with corticosteroids: 8 Nutritional support only: 17 Pentoxifylline: 10 FMT: 16	(1) The proportions of patients surviving at the end of 1 and 3 months in the steroids, nutrition, pentoxifylline, and FMT group were 63%, 47%, 40%, and 75% (*p* = 0.179) and 38%, 29%, 30%, and 75% (*p* = 0.036), respectively. (2) Compared with FMT, relative risk and hazard ratios for death were higher in all the other groups.
	[67]	Control: 10 FMT: 10	(1) This phase 1 trial shows that FMT is safe and associated with short-term reduction in alcohol craving and consumption with favorable microbial changes versus placebo in patients with alcohol-related cirrhosis with alcohol misuse. (2) There was also a reduction in AUD-related events over 6 months in patients assigned to FMT.
PSC	[90]	FMT:10 without controls	(1) Abundance of engrafter operational taxonomic units in patients post-FMT correlated with decreased ALP levels (*p* = 0.02). (2) 30% (3/10) experienced a ≥50% decrease in ALP levels. (3) The diversity increased in all patients post-FMT, as early as week 1 (*p* < 0.01).

ALD, alcoholic liver disease; ALP, alkaline phosphatase; ALT, alanine aminotransferase; AUD, alcohol use disorder; AVT, antiviral therapy; DNA, deoxyribonucleic acid; FMT, fecal microbiota transplantation; HBeAg, HBV e-antigen; HBsAg, hepatitis B surface antigen; HBV, hepatitis B virus; NAFLD, non-Alcoholic fatty liver disease; PSC, primary sclerosing cholangitis.

### 4.6. Hepatocellular Carcinoma (HCC)

HCC is an aggressive tumor, which is frequently diagnosed at a late stage with a median survival of about 6–20 months. By 2025, over one million cases will be globally affected by HCC [91]. LT has become a standard treatment for patients with early stage HCC in several countries [92]. However, for those patients with advanced HCC whose number and size of tumor beyond Milan criteria, the 5-year survival rate after LT remains poor [93]. Over 80% of HCC cases are associated with liver cirrhosis, representing inflammation and hepatocellular proliferation [94]. A study demonstrated that bacteria derived from gut might play a role in the recurrence of cirrhosis and HCC [95]. In addition, the latest animal studies demonstrated that gut microbiota and its metabolites could directly affect intrahepatic and peripheral inflammatory and immune responses in HCC [96,97], while immune response in HCC influences the clinical course and overall survival of the disease, mainly by impairing the functions of T cells and regulatory T cells (Tregs). To date, several investigations revealed that the gut microbiota could promote the development of HCC via the gut–liver axis [95,98,99]. Therefore, modulation of gut microbiota is deemed as a method to prevent HCC [100].

Some clinical trials related to gut microbiota in HCC have been performed or are ongoing. These studies mainly involve administration of probiotics for the treatment of HCC (Identifiers: NCT02021253, NCT05178524, and NCT03853928). However, to our knowledge, no clinical study has assessed the applicability of FMT for HCC. Only Baruch et al. reported the first human clinical trial where they found how treatment with FMT was associated with favorable changes in gene expression profiles and immune cell infiltrates in the tumor microenvironment [101]. These data indicated the benefits of FMT for the treatment of HCC [102].

## 5. Clinical Value of Fecal Microbiota Transplantation (FMT) for Liver Transplantation (LT) Recipients

It is widely accepted that LT is still the only therapeutic option for patients with end-stage liver disease, acute liver failure, and HCC [103]. Over the recent decades, LT has been used as a mature and conventional surgical method for liver diseases [104]. However, patients receiving LT are at a particularly higher risk of infection, such as CDI [105], cytomegalovirus (CMV) infection [106], fungi infection, recurrent HBV infection, etc. A previous cohort study demonstrated that about 19% of deaths occurred at five years after LT were related to various sources of infection [103,107]. That is mainly due to administration of immunosuppressive agents after LT attenuates immune surveillance, enabling pathogens to evade natural immunity and facilitate infection. Furthermore, pre-transplant infection and some other risk factors are also associated with post-LT infection [108]. In addition, several studies demonstrated that the types of gut microbiota may significantly change after LT [109,110,111]. Hence, restoring the gut microbiota balance by FMT may be particularly critical for LT recipients. For instance, Schneider et al. reported a case of successful FMT in a LT recipient with severe CDI that was complicated with acute kidney injury [112]. Furthermore, the safety of FMT in immunocompromised patients has been demonstrated in a meta-analysis of 44 studies [113]. Therefore, FMT may be a potential therapeutic method for CDI after LT. However, to our knowledge, no clinical study has yet assessed the applicability of FMT for infectious diseases.

## 6. Conclusions and Future Perspectives

Human biology is contextual on the coexisting microorganisms, with the majority living in the digestive tract, influencing various physiological functions [55]. The gut–liver axis has shown a mutual association between the intestine and liver. Hence, the close interaction between gut and liver may be a very important factor in the pathogenesis of liver diseases. In addition, a growing body of evidence demonstrated that FMT is a novel approach to reconstruct the intestinal microecological balance, therefore, FMT has been gradually and widely utilized in the treatment of several liver diseases. In this review, the clinical progress of FMT in the treatment of liver diseases was briefly summarized. However, it is noteworthy that standards related to quality and safety control of microbiota have not been widely accepted. Consequently, FMT and other microbiota-based treatments are still in the preclinical stage for patients with some liver diseases, such as AIH and HCC, thereby deserving further clinical investigation. Moreover, restoring the gut microbiota balance by FMT may be particularly critical for LT recipients.

## Data Availability

Not applicable.

## Abbreviations

alcoholic liver disease (ALD), non-Alcoholic fatty liver disease (NAFLD), primary sclerosing cholangitis (PSC), hepatocellular carcinoma (HCC), fecal microbiota transplantation (FMT), liver transplant (LT), gastrointestinal (GI), clostridium difficile infection (CDI), hepatic encephalopathy (HE), hepatitis B virus (HBV), hepatitis B surface antigen (HBsAg), HBV e-antigen (HBeAg), chronic hepatitis B (CHB), entecavir (ETV), tenofovir disoproxil fumarate (TDF), tenofovir, alafenamide (TAF), nonalcoholic steatohepatitis (NASH), type 2 diabetes mellitus (T2D), high-fat diet (HFD), autoimmune hepatitis (AIH), follicular regulatory T (TFR), helper T (TFH), Toll-like receptor 4 (TLR4), inflammatory bowel disease (IBD), fucosyltransferase 2 (FUT2), germ-free multidrug resistance 2 (mdr2), alkaline phosphatase (ALP), regulatory T cells (Tregs), cytomegalovirus (CMV), acute kidney injury (AKI).
